# **ϵ**-Henig Saddle Points and Duality of Set-Valued Optimization Problems in Real Linear Spaces

**DOI:** 10.1155/2013/403642

**Published:** 2013-10-03

**Authors:** Zhi-Ang Zhou

**Affiliations:** College of Mathematics and Statistics, Chongqing University of Technology, Chongqing 400054, China

## Abstract

We study **ϵ**-Henig saddle points and duality of set-valued optimization problems in the setting of real linear spaces. Firstly, an equivalent characterization of **ϵ**-Henig saddle point of the Lagrangian set-valued map is obtained. Secondly, under the assumption of the generalized cone subconvexlikeness of set-valued maps, the relationship between the **ϵ**-Henig saddle point of the Lagrangian set-valued map and the **ϵ**-Henig properly efficient element of the set-valued optimization problem is presented. Finally, some duality theorems are given.

## 1. Introduction

Saddle points and duality of set-valued optimization, which have close relationship to the generalized convexity of set-valued maps and the efficiency of solutions of set-valued optimization, are two important topics in optimization theory. Recently, many new researches involving saddle points and duality of set-valued optimization have appeared in the literature. Li [1] introduced Benson proper saddle point of the Lagrangian set-valued maps for a set-valued optimization problem in locally convex spaces and established saddle point theorems and duality theorems under the assumption of the cone subconvexlikeness of set-valued maps. Zhao and Rong [2] and Li et al. [3] investigated *ϵ*-strict saddle points and duality of set-valued optimization problems under the assumption of cone convexlikeness and ic-cone convexlikeness of set-valued maps, respectively. Xia and Qiu [4] obtained saddle point theorems and duality theorems of set-valued optimization problems in the sense of super efficiency under the nearly subconvexlikeness of set-valued maps.

However, in the above mentioned references, saddle points and duality of set-valued optimization were studied in locally convex spaces. How to generalize saddle point theorems and duality theorems of set-valued optimization from locally convex spaces to linear spaces is interesting. Adán and Novo [5] studied saddle points and duality for convexlike vector optimization problems in real linear spaces. In the *ϵ*-global prober efficiency, Zhou et al. [6] introduced the concept of the *ϵ*-global prober saddle point and obtained the relationships between the *ϵ*-global proper saddle points of Lagrangian set-valued maps and the  *ϵ*-global properly efficient element of set-valued optimization problems.

This paper is a continuation of the research work [7]. The aim of this paper is to investigate *ϵ*-Henig proper saddle points and duality of set-valued optimization problems in linear spaces. This paper is organized as follows. In Section 2, some preliminaries, including notations and lemmas, are given. In Section 3, we introduce a new notion of *ϵ*-Henig proper saddle point of the Lagrangian set-valued map in linear spaces and obtain several saddle point theorems. In Section 4, we present three duality theorems including *ϵ*-weak duality, *ϵ*-converse duality, and *ϵ*-strong duality.

## 2. Preliminaries

Let *X* be a real linear space, and let *Y* and *Z* be two real ordered linear spaces. 0 denotes the zero element of every space. Let *K* be a nonempty subset in *Y*. The generated cone of *K* is defined as cone⁡(*K*): = {*λk* | *k* ∈ *K*, *λ* ≥ 0}. *K* is called a cone if and only if *λK*⊆*K* for any *λ* ≥ 0. A cone *K* is said to be pointed if and only if *K*∩(−*K*) = {0}.  *K* is said to be nontrivial if and only if *K* ≠ {0} and *K* ≠ *Y*.

The algebraic dual of *Y* and *Z* is denoted by Y* and Z*, respectively. Let *C* and *D* be two nontrivial pointed convex cones in *Y* and *Z*, respectively. The algebraic dual cone *C* ^+^ and strictly algebraic dual cone *C* ^+*i*^ of *C* are, respectively, defined as

(1) $$\begin{array}{c} C^{+}:=\left\{y^{*}\in Y^{*}\;|\;\langle y,y^{*}\rangle ⩾0,\;\;\forall y\in C\right\},\\ C^{+i}:=\left\{y^{*}\in Y^{*}\;|\;\langle y,y^{*}\rangle >0,\;\;\forall y\in C\setminus \left\{0\right\}\right\}, \end{array}$$

where 〈*y*, y*〉 denotes the value of the linear functional y* at the point *y*. The meaning of *D* ^+^ is similar to that of *C* ^+^.

**Definition (see [8])**

Let *K* be a nonempty subset in *Y*. The algebraic interior of *K*  is the set

(2) cor(K):={k∈K ∣ ∀h∈Y,  ∃λ′>0,  ∀λ∈[0,λ′],  k+λh∈K}.

**Definition 2 (see [9])**

Let *K* be a nonempty subset in *Y*. *K* is balanced if and only if, for all  *x* ∈ *K*, for all *λ* ∈ [−1,1], *λk* ∈ *K*.  *K* is called absorbent if and only if 0 ∈ cor(*K*).

**Definition (see [10])**

Let *K* be a nonempty subset in *Y*. The vector closure of *K* is the set

(3) vcl(K):={k∈Y ∣ ∃h∈Y,  ∀λ′>0,  ∃λ∈]0,λ′], k+λh∈K}.

Following Adán and Novo [10], *K* is vectorially closed (v-closed) if vcl(*K*) = *K*.

**Definition (see [7])**

Let  *B*  be a nonempty convex subset in *Y*. *B* is a base of *C* if and only if *C* = cone⁡(*B*) and there exists a balanced, absorbent, and convex set *V* such that 0 ∉ *B* + *V* in *Y*. Write *C*_*V*_(*B*): = cone⁡(*V* + *B*).

From now on, we suppose that cor(*C*) × cor(*D*) ≠ *∅* and *B* is a base of *C*. We recall a notion of  *ϵ*-Henig properly efficient point introduced by Zhou et al. [7] in linear spaces.

**Definition (see [7])**

Let *K*⊆*Y* and *ϵ* ∈ *C*. $\bar{y}\in K$ is called an *ϵ*-Henig properly minimal efficient point with respect to *B* (denoted by $\bar{y}\in \epsilon$-*H*_min⁡_(*K*, *B*)) if and only if there exists a balanced, absorbent, and convex set *V* with 0 ∉ *B* + *V* such that $\mathrm{cone}(K-\bar{y}+\epsilon )\cap (-C_{V}(B))=\{0\}.\;\bar{y}\in K$ is called an *ϵ*-Henig properly maximal efficient point with respect to  *B*  (denoted by $\bar{y}\in \epsilon \text{-}H_{\max}(K,B)$) if and only if there exists a balanced, absorbent, and convex set *V* with 0 ∉ *B* + *V* such that $\mathrm{cone}(K-\bar{y}-\epsilon )\cap C_{V}(B)=\{0\}$.

Let *A* be a nonempty set in *X* and *F* : *A*⇉*Y* and *G* : *A*⇉*Z* be two set-valued maps on *A*. Write *F*(*A*): = ⋃_*x*∈*A*_ *F*(*x*), 〈*F*(*x*), y*〉: = {〈*y*, y*〉∣*y* ∈ *F*(*x*)}, and 〈*F*(*A*), y*〉: = ⋃_*x*∈*A*_〈*F*(*x*), y*〉. The meanings of *G*(*A*), 〈*G*(*x*), z*〉, and 〈*G*(*A*), z*〉 are similar to those of *F*(*A*), 〈*F*(*x*), y*〉, and 〈*F*(*A*), y*〉, respectively.

**Definition (see [11])**

A set-valued map *F* : *A*⇉*Y* is called generalized *C*-subconvexlike on *A* if and only if cone⁡(*F*(*A*)) + cor(*C*)  is a convex set in *Y*.

**Lemma (see [6])**

Let *Z* be a linear space, and let *M*, *N*⊆*Z* be two nonempty sets such that *M* − *N* is a convex set in *Z*. If  *cor*(*M* − *N*) ≠ *∅* and 0 ∉ *vcl*(*M* − *N*), then there exists z* ∈ Z*∖{0} such that sup⁡_*z*_2_∈*N*_〈*z*_2_, z*〉<inf⁡_*z*_1_∈*M*_〈*z*_1_, z*〉.

## 3. *ϵ*-Henig Saddle Points

In this section, we will establish approximate saddle point theorems of set-valued optimization problems in the sense of *ϵ*-Henig proper efficiency.

Let *F* : *A*⇉*Y* and *G* : *A*⇉*Z* be two set-valued maps on *A*. We consider the following vector optimization problem with set-valued maps:

(4) $$\begin{array}{c} \left(\text{VP}\right)\min F\left(x\right)\;\;\text{subject}\;\text{to}\;G\left(x\right)\cap \left(-D\right)\neq \emptyset . \end{array}$$

The feasible set of (VP) is defined by *S* : = {*x* ∈ *A* | *G*(*x*)∩(−*D*) ≠ *∅*}.  

**Definition 8 (see [7])**

Let *ϵ* ∈ *C*. $\bar{x}\in S$ is called an *ϵ*-Henig properly efficient solution of (VP) if and only if there exists $\bar{y}\in F(\bar{x})$ such that $\bar{y}\in \epsilon$-*H*_min⁡_(*F*(*S*), *B*). The pair $\left(\bar{x},\bar{y}\right)$ is called an *ϵ*-Henig properly efficient element of (VP).

We denote by *L*(*Z*, *Y*) the set of all linear operators from *Z* to *Y*. A subset *L* ^+^(*Z*, *Y*) of *L*(*Z*, *Y*) is defined as *L* ^+^(*Z*, *Y*): = {*T* ∈ *L*(*Z*, *Y*) | *T*(*D*)⊆*C*}. The Lagrangian set-valued map of (VP) is defined by

(5) $$\begin{array}{c} L\left(x,T\right):=F\left(x\right)+T\left(G\left(x\right)\right),\;\forall \left(x,T\right)\in A\times L^{+}\left(Z,Y\right). \end{array}$$

Consider the following unconstrained vector optimization problem with set-valued maps:

(6) $$\begin{array}{c} {\left(\text{UVP}\right)}_{T}\;\;\min L\left(x,T\right)\;\;\;\;\text{subject}\;\;\text{to}\;\;\left(x,T\right)\in A\times L^{+}\left(Z,Y\right). \end{array}$$

**Lemma 9 (see [7])**

Let *ϵ* ∈ *C*,  $\bar{x}\in S$, and $\bar{y}\in F(\bar{x})$. If there exists $\bar{T}\in L^{+}(Z,Y)$ such that $\left(\bar{x},\bar{y}\right)$ is an *ϵ*-Henig properly efficient element of ${\left(UVP\right)}_{\bar{T}}$, then $\left(\bar{x},\bar{y}\right)$ is an *ϵ*-Henig properly efficient element of (VP).

Now, we will introduce a new notion called *ϵ*-Henig proper saddle point of the Lagrangian set-valued map *L*(*x*, *T*) in linear spaces.

**Definition 10**

$\left(\bar{x},\bar{T})\in A\times L^{+}(Z,Y\right)$ is called an  *ϵ*-Henig proper saddle point of the Lagrangian set-valued map *L*(*x*, *T*) if and only if

(7) L(x¯,T¯)∩ϵ-Hmin⁡(⋃x∈AL(x,T¯),B)∩ϵ-Hmax⁡(⋃T∈L+(Z,Y)L(x¯,T),B)≠∅.

The following proposition is an important equivalent characterization for an  *ϵ*-Henig proper saddle point of the Lagrangian set-valued map *L*(*x*, *T*).

**Proposition 11**

Let *D* be v-closed and *ϵ* ∈ *C*. Then, $\left(\bar{x},\bar{T})\in A\times L^{+}(Z,Y\right)$ is an  *ϵ*-Henig proper saddle point of the Lagrangian set-valued map $L(x,\bar{T})$ if and only if there exist $\bar{y}\in F(\bar{x})$,  $\bar{z}\in G(\bar{x})$, and a balanced, absorbent, and convex set *V* with 0 ∉ *B* + *V* such that

- $\bar{y}+\bar{T}(\bar{z})\in \epsilon \text{-}H_{\text{min }}({⋃}_{x\in A}L(x,\bar{T}),B)$ *;*
- $G(\bar{x})\subseteq -D$ *;*
$-\bar{T}(\bar{z})\in C\setminus (\epsilon +C\setminus \{0\})$ *;*
- $cone(F(\bar{x})-\bar{y}-\bar{T}(\bar{z})-\epsilon )\cap C_{V}(B)=\{0\}$.

**Proof**

*Necessity*. Let $\left(\bar{x},\bar{T}\right)$ be an *ϵ*-Henig proper saddle point of *L*(*x*, *T*). Then, there exist $\bar{y}\in F(\bar{x})$ and $\bar{z}\in G(\bar{x})$ such that

(8) $$\begin{array}{c} \bar{y}+\bar{T}\left(\bar{z}\right)\in \epsilon \text{-}H_{\min}\left(\underset{x\in A}{⋃}L\left(x,\bar{T}\right),B\right), \end{array}$$

(9) $$\begin{array}{c} \bar{y}+\bar{T}\left(\bar{z}\right)\in \epsilon \text{-}H_{\max}\left(\underset{T\in L^{+}(Z,Y)}{⋃}L\left(\bar{x},T\right),B\right). \end{array}$$

Equation (8) shows that (i) holds. By (9), there exists a balanced, absorbent, and convex set *V* with 0 ∉ *B* + *V* such that

(10) $$\begin{array}{c} \mathrm{cone}\left(\underset{T\in L^{+}\left(Z,Y\right)}{⋃}L\left(\bar{x},T\right)-\bar{y}-\bar{T}\left(\bar{z}\right)-\epsilon \right)\cap C_{V}\left(B\right)=\left\{0\right\}. \end{array}$$

Taking *T* = 0 in (10), we obtain

(11) $$\begin{array}{c} \mathrm{cone}\left(F\left(\bar{x}\right)-\bar{y}-\bar{T}\left(\bar{z}\right)-\epsilon \right)\cap C_{V}\left(B\right)=\left\{0\right\}. \end{array}$$

Therefore, (iv) holds. Since $\bar{y}\in F(\bar{x})$ and *V* is absorbent in *Y*, it follows from (11) that

(12) $$\begin{array}{c} \mathrm{cone}\left(\bar{T}\left(\bar{z}\right)+\epsilon \right)\cap \left(-B\right)=\emptyset . \end{array}$$

Because cone⁡(*B*) = *C*, it follows from (12) that $\mathrm{cone}(\bar{T}(\bar{z})+\epsilon )\;\cap \;(-C\setminus \{0\})=\emptyset$. Clearly, $(\bar{T}(\bar{z})+\epsilon )\cap (-C\setminus \{0\})=\emptyset$. Therefore,

(13) $$\begin{array}{c} -\bar{T}\left(\bar{z}\right)\notin \epsilon +C\setminus \left\{0\right\}. \end{array}$$

We assert that $-\bar{z}\in D$. Otherwise, by Lemma 7, it is easy to prove (see the proof of Proposition  4.1 in [6]) that there exists *z*_1_* ∈ *D* ^+^∖{0} such that $\langle \bar{z},z_{1}^{*}\rangle >0$. Taking *b*_1_ ∈ *B*, we define a vector-valued map *T*_1_ : *Z* → *Y* as follows:

(14) $$\begin{array}{c} T_{1}\left(z\right)=\frac{\langle z,z_{1}^{*}\rangle }{\langle \bar{z},z_{1}^{*}\rangle }\left(b_{1}+\epsilon \right)+\bar{T}\left(z\right),\;\forall z\in Z. \end{array}$$

Clearly, *T*_1_ ∈ *L* ^+^(*Z*, *Y*) and $T_{1}(\bar{z})-\bar{T}(\bar{z})-\epsilon =b_{1}\in C_{V}(B)$. On the other hand, $T_{1}(\bar{z})-\bar{T}(\bar{z})-\epsilon \in \mathrm{cone}({⋃}_{T\in L^{+}(Z,Y)}L(\bar{x},T)-\bar{y}-\bar{T}(\bar{z})-\epsilon )$. Since 0 ∉ *B* + *V*, *b*_1_ ≠ 0. Therefore,

(15) $$\begin{array}{c} \mathrm{cone}\left(\underset{\text{T}\in L^{+}\left(Z,Y\right)}{⋃}L\left(\bar{x},T\right)-\bar{y}-\bar{T}\left(\bar{z}\right)-\epsilon \right)\cap C_{V}\left(B\right)\neq \left\{0\right\}, \end{array}$$

which contradicts (10). Hence,$\;-\bar{z}\in D$. Since $\bar{T}\in L^{+}(Z,Y)$, we have

(16) $$\begin{array}{c} -\bar{T}\left(\bar{z}\right)\in C. \end{array}$$

It follows from (13) and (16) that (iii) holds. We assert that $G(\bar{x})\subseteq -D$. Otherwise, there exists $z_{1}\in G(\bar{x})$ such that *z*_1_ ∉ −*D*. Similar to the above proof, there exists *z*_2_* ∈ *D* ^+^∖{0} such that 〈*z*_1_, *z*_2_*〉>0. Taking *b*_2_ ∈ *B*, we define a vector-valued map *T*_2_ : *Z* → *Y* as follows:

(17) $$\begin{array}{c} T_{2}\left(z\right)=\frac{\langle z,z_{2}^{*}\rangle }{\langle z_{1},z_{2}^{*}\rangle }\left(b_{2}+\epsilon \right),\;\forall z\in Z. \end{array}$$

Clearly,

(18) $$\begin{array}{c} T_{2}\in L^{+}\left(Z,Y\right), \end{array}$$

(19) $$\begin{array}{c} T_{2}\left(z_{1}\right)-\bar{T}\left(\bar{z}\right)-\epsilon =b_{2}-\bar{T}\left(\bar{z}\right)\in B+C\subseteq C\setminus \left\{0\right\}. \end{array}$$

Since $\bar{y}\in F(\bar{x})$ and $z_{1}\in G(\bar{x})$, it follows from (10) and (18) that

(20) $$\begin{array}{c} \mathrm{cone}\left(T_{2}\left(z_{1}\right)-\bar{T}\left(\bar{z}\right)-\epsilon \right)\cap C_{V}\left(B\right)=\left\{0\right\}. \end{array}$$

By (20), it is easy to check that $T_{2}(z_{1})-\bar{T}(\bar{z})-\epsilon \notin C\setminus \{0\}$, which contradicts (19). Therefore, (ii) holds.

*Sufficiency*. Since $\bar{y}+\bar{T}(\bar{z})\in L(\bar{x},\bar{T})$, by condition (i), we only prove that

(21) $$\begin{array}{c} \bar{y}+\bar{T}\left(\bar{z}\right)\in \epsilon \text{-}H_{\max}\left(\underset{T\in L^{+}\left(Z,Y\right)}{⋃}\text{L}\left(\bar{x},T\right),B\right). \end{array}$$

We assert

(22) $$\begin{array}{c} \mathrm{cone}\left(\underset{T\in L^{+}\left(Z,Y\right)}{⋃}L\left(\bar{x},T\right)-\bar{y}-\bar{T}\left(\bar{z}\right)-\epsilon \right)\cap C_{V}\left(B\right)=\left\{0\right\}. \end{array}$$

Otherwise, there exists *r*_1_ > 0,   *r*_2_ > 0,  *T*_3_ ∈ *L* ^+^(*Z*, *Y*),   $y^{\prime }\in F(\bar{x})$,  $z^{\prime }\in G(\bar{x})$,  *v* ∈ *V*,  and  *b*_3_ ∈ *B* such that $r_{1}(y^{\prime }+T_{3}(z^{\prime })-\bar{y}-\bar{T}(\bar{z})-\epsilon )=r_{2}(v+b)$. Clearly,

(23) $$\begin{array}{c} r_{1}\left(y^{\prime }-\bar{y}-\bar{T}\left(\bar{z}\right)-\epsilon \right)=r_{2}\left(v+b_{3}\right)-r_{1}T_{3}\left(z^{\prime }\right). \end{array}$$

By condition (ii),  *z*′ ∈ −*D*. Since *T*_3_ ∈ *L* ^+^(*Z*, *Y*),  −*T*_3_(*z*′) ∈ *C*. Therefore, there exist *r*_3_ ≥ 0 and *b*_4_ ∈ *B* such that

(24) $$\begin{array}{c} -T_{3}\left(z^{\prime }\right)=r_{3}b_{4}. \end{array}$$

It follows from (23) and (24) that

(25) r1(y′−y¯−T¯(z¯)−ϵ)  =r2(v+b3)+r1r3b4  =r2v+(r2b3+r1r3b4)=(r2+r1r3)   ×[r2r2+r1r3v+(r2r2+r1r3b3+r1r3r2+r1r3b4)].

Since 0 < *r*_2_/(*r*_2_ + *r*_1_ *r*_3_) ≤ 1, it follows from the balance of  *V*  that

(26) $$\begin{array}{c} \frac{r_{2}}{r_{2}+r_{1}r_{3}}v\in V. \end{array}$$

It follows from the convexity of *B* that

(27) $$\begin{array}{c} \frac{r_{2}}{r_{2}+r_{1}r_{3}}b_{3}+\frac{r_{1}r_{3}}{r_{2}+r_{1}r_{3}}b_{4}\in B. \end{array}$$

Since 0 ∉ *V* + *B* and *r*_2_ + *r*_1_ *r*_3_ > 0, it follows from (25)–(27) that

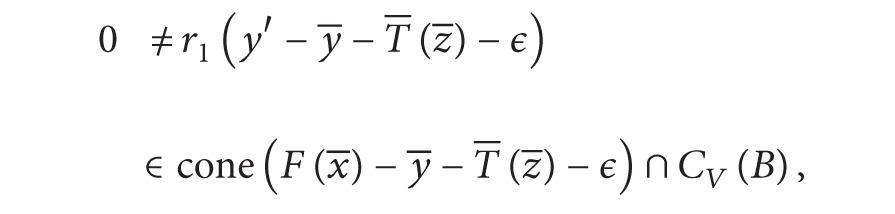
(28)
which contradicts condition (iv). Therefore, (22) holds. Thus, (21) holds.

**Remark**

According to Theorem 1 in [12], the notion of *ϵ*-strictly efficient point is equivalent to the notion of *ϵ*-Henig properly efficient point in locally convex spaces. Moreover, the generalized subconvexlikeness of the set-valued map *F* is equivalent to ic-cone convexlikeness of the set-valued map *F* introduced by Sach [13] when the topological interior int⁡(*C*) ≠ *∅*. Therefore, Proposition 11 generalizes Proposition 5.1 in [3] from locally convex spaces to real linear spaces.

**Theorem**

Let *D* be v-closed and *ϵ* ∈ *C*. If $\left(\bar{x},\bar{T})\in A\times L^{+}(Z,Y\right)$ is an *ϵ*-Henig proper saddle point of the Lagrangian set-valued map *L*(*x*, *T*), then there exist $\bar{y}\in F(\bar{x})$ and  $\bar{z}\in G(\bar{x})$ such that $\left(\bar{x},\bar{y}\right)$ is an $\bar{\epsilon }$-Henig properly efficient element of (VP), where $\bar{\epsilon }=\epsilon -\bar{T}(\bar{z})$.

**Proof**

Since $\left(\bar{x},\bar{T})\in A\times L^{+}(Z,Y\right)$ is an *ϵ*-Henig proper saddle point of the Lagrangian set-valued map *L*(*x*, *T*), it follows from Proposition 11 that there exist $\bar{y}\in F(\bar{x})$,  $\bar{z}\in G(\bar{x})$, and a balanced, absorbent, and convex set *V* with  0 ∉ *B* + *V* such that conditions (i)–(iv) hold. By condition (ii), $\bar{x}\in S$. By condition (iii),  $\bar{\epsilon }=\epsilon -\bar{T}(\bar{z})\in C+C\setminus (\epsilon +C\setminus \{0\})\subseteq C$. We assert that $\left(\bar{x},\bar{y}\right)$ is an $\bar{\epsilon }$-Henig properly efficient element of (VP). Otherwise, for any balanced, absorbent, and convex set *U* with $0\notin B\;+\;U,\mathrm{cone}(F(S)\;-\;\bar{y}\;+\;\bar{\epsilon })\cap (-C_{U}(B))\neq \{0\}$. Therefore, there exist *r*_1_ > 0,  *r*_2_ > 0,  *x*_1_ ∈ *S*,  *y*_1_ ∈ *F*(*S*),  *b*_1_ ∈ *B*, and  *u*_1_ ∈ *U* such that

(29) $$\begin{array}{c} r_{1}\left(y_{1}-\bar{y}+\bar{\epsilon }\right)=-r_{2}\left(b_{1}+u_{1}\right). \end{array}$$

It follows from (29) that

(30) r1(y1+T¯(z)+ϵ−(y¯+T¯(z¯)))=−r2(b1+u1)+r1T¯(z),                     ∀z∈G(x¯)∩(−D).

Since $\bar{T}\in L^{+}(Z,Y)$, for any $z\in G(\bar{x})\cap (-D)$, it follows from (30) that there exists *c* ∈ *C* such that

(31) $$\begin{array}{c} r_{1}\left(y_{1}+\bar{T}\left(z\right)+\epsilon -\left(\bar{y}+\bar{T}\left(\bar{z}\right)\right)\right)=-r_{2}\left(b_{1}+u_{1}\right)-r_{1}c. \end{array}$$

Because *B* is a base of *C*, there exist *r*_3_ ≥ 0 and *b*_2_ ∈ *B* such that *c* = *r*_3_ *b*_2_. By (31), we obtain

(32) r1(y1+T¯(z)+ϵ−(y¯+T¯(z¯)))  =−r2(b1+u1)−r1r3b2  =−[r2u1+(r2b1+r1r3b2)]  =−(r2+r1r3)   ×[r2r2+r1r3u1     +(r2r2+r1r3b1+r1r3r2+r1r3b2)]∈−CU(B).

Clearly, $r_{1}\left(y_{1}+\bar{T}\left(z\right)+\epsilon -\left(\bar{y}+\bar{T}\left(\bar{z}\right)\right)\right)\neq 0$ $\text{for}\;\text{all}\;\;z\in G(\bar{x})\cap (-D)$. Therefore, we obtain

(33) $$\begin{array}{c} \mathrm{cone}\left(\underset{x\in A}{⋃}L\left(x,\bar{T}\right)+\epsilon -\left(\bar{y}+\bar{T}\left(\bar{z}\right)\right)\right)\cap \left(-C_{U}\left(B\right)\right)\neq \left\{0\right\}, \end{array}$$

which contradicts $\bar{y}+\bar{T}(\bar{z})\in \epsilon$-$H_{\min}({⋃}_{x\in A}L(x,\bar{T}),B)$. Hence, $\left(\bar{x},\bar{y}\right)$ is an $\bar{\epsilon }$-Henig properly efficient element of (VP).

**Remark**

Comparing Theorem 13 with Theorem  4.1 in [6], the notion of *ϵ*-global proper efficiency has been replaced by the notion of *ϵ*-Henig proper efficiency and the condition $0\in G(\bar{x})$ has been dropped.

In order to obtain sufficient conditions of  *ϵ*-Henig proper saddle point under the assumption of the generalized cone subconvexlikeness, we need the following lemma.

**Lemma (see [7])**

Let *ϵ* ∈ *C*,  $\bar{x}\in S$, and $0\in G(\bar{x})$. Suppose that the following conditions hold:

- $\left(\bar{x},\bar{y}\right)$ is an *ϵ*-Henig properly efficient element of (VP);
- $\bar{I}(x)$ is generalized *C* × *D*-subconvexlike on *A*, where $\bar{I}(x)=(F(x)-\bar{y}+\epsilon )\times G(x)$;
- *vcl*(*cone*(*G*(*A*) + *D*)) = *Z*.

Then, there exists $\bar{T}\in L^{+}(Z,Y)$ such that $\left(\bar{x},\bar{y}\right)$ is an  *ϵ*-Henig properly efficient element of ${\left(UVP\right)}_{\bar{T}}$.

By Lemma 15, we easily obtain the following theorem involving the generalized cone subconvexlikeness of set-valued maps.

**Theorem**

Let *D* be v-closed, *ϵ* ∈ *C*,  $\bar{x}\in S$, and $0\in G(\bar{x})$. Suppose that the following conditions hold:

- $\left(\bar{x},\bar{y}\right)$ is an  *ϵ*-Henig properly efficient element of (VP);
- $\bar{I}(x)$ is generalized  *C* × *D*-subconvexlike on *A*, where $\bar{I}(x)=(F(x)-\bar{y}+\epsilon )\times G(x)$;
- *vcl*(*cone*(*G*(*A*) + *D*)) = *Z*;
- $\bar{y}\in \epsilon$-$H_{\text{max }}({⋃}_{T\in L^{+}(Z,Y)}L(\bar{x},T),C)$.

Then, there exists $\bar{T}\in L^{+}(Z,Y)$ such that $\left(\bar{x},\bar{T}\right)$ is an *ϵ*-Henig proper saddle point of  *L*.

## 4. *ϵ*-Duality

In this section, we will give several duality theorems characterized by *ϵ*-Henig proper efficiency of set-valued optimization problems in linear spaces.

**Definition**

Let *ϵ* ∈ *C* and let *B* be a base of *C*. The set-valued map Φ : *L* ^+^(*Z*, *Y*)⇉*Y*, defined by Φ(*T*) = *ϵ*-*H*_min⁡_(⋃_*x*∈*A*_ *L*(*x*, *T*), *B*), is called an *ϵ*-Henig properly dual map of (VP).

Now, we construct the following duality problem of the primal problem (VP):

(34) $$\begin{array}{c} \left(\text{VD}\right)\;\;\max\underset{T\in L^{+}\left(Z,Y\right)}{⋃}\Phi \left(T\right). \end{array}$$

**Definition 18**

Let *ϵ* ∈ *C*.$\;\bar{y}\in {⋃}_{T\in L^{+}(Z,Y)}\Phi (T)$ is called an  *ϵ*-efficient point of (VD) if and only if

(35) $$\begin{array}{c} \left(\underset{T\in L^{+}\left(Z,Y\right)}{⋃}\Phi \left(T\right)-\bar{y}-\epsilon \right)\cap \left(C\setminus \left\{0\right\}\right)=\emptyset . \end{array}$$

**Theorem 19 (*ϵ*-weak duality)**

Let *ϵ* ∈ *C*,  $\bar{x}\in S$, and $\bar{y}\in {⋃}_{T\in L^{+}(Z,Y)}\Phi (T)$. Then, $(\bar{y}-F(\bar{x})-\epsilon )\cap (C\setminus \{0\})=\emptyset$.

**Proof**

Since $\bar{y}\in {⋃}_{T\in L^{+}(Z,Y)}\Phi (T)$, there exists $\bar{T}\in L^{+}(Z,Y)$ such that $\bar{y}\in \Phi (\bar{T})$. Clearly, $\bar{y}\in \epsilon$-$H_{\min}({⋃}_{x\in A}L(x,\bar{T}),B)$. Thus, there exists a balanced, absorbent, and convex set *V* with 0 ∉ *B* + *V* such that $\mathrm{cone}({⋃}_{x\in A}L(x,\bar{T})-\bar{y}+\epsilon )\cap (-C_{V}(B))=\{0\}$. It is easy to check *C*∖{0}⊆*C*_*V*_(*B*)∖{0}. Therefore, $(\bar{y}-{⋃}_{x\in A}L(x,\bar{T})-\epsilon )\cap (C\setminus \{0\})=\emptyset$. Since $\bar{x}\in S\subseteq A,(\bar{y}-F(\bar{x})-\bar{T}(G(\bar{x}))-\epsilon )\cap (C\setminus \{0\})=\emptyset$. Because $\bar{x}\in S$, there exists $\bar{z}\in G(\bar{x})\cap (-D)$ such that $(\bar{y}-F(\bar{x})-\bar{T}(\bar{z})-\epsilon )\cap (C\setminus \{0\})=\emptyset$. Since $\bar{T}(\bar{z})\in C$, it is easy to check that $(\bar{y}\;-\;F(\bar{x})\;-\;\epsilon )\cap (C\setminus \{0\})=\emptyset$.

**Theorem (*ϵ*-converse duality)**

Let *ϵ* ∈ *C* and$\;\bar{x}\in S$. If $\bar{y}\in F(\bar{x})\cap {⋃}_{T\in L^{+}(Z,Y)}\Phi (T)$ and $0\in G(\bar{x})$, then $\left(\bar{x},\bar{y}\right)$ is an *ϵ*-Henig properly efficient element of (VP) and $\bar{y}$ is *ϵ*-efficient point of (VD).

**Proof**

Since $\bar{y}\in F(\bar{x})\cap {⋃}_{T\in L^{+}(Z,Y)}\Phi (T)$, there exists $\bar{T}\in L^{+}(Z,Y)$ such that $\bar{y}\in \Phi (\bar{T})$. It follows from $0\in G(\bar{x})$ and the definition of  Φ  that$\;(\bar{x},\bar{y})\;$is an  *ϵ*-Henig properly efficient element of ${\left(\text{UVP}\right)}_{\bar{T}}$. According to Lemma 9,$\;(\bar{x},\bar{y})\;$is an  *ϵ*-Henig properly efficient element of (VP). Because $\bar{x}\in S$ and $\bar{y}\in {⋃}_{T\in L^{+}(Z,Y)}\Phi (T)$, using Theorem 19, we have $({⋃}_{T\in L^{+}(Z,Y)}\Phi (T)-F(\bar{x})-\epsilon )\cap (C\setminus \{0\})=\emptyset$. Clearly, $({⋃}_{T\in L^{+}(Z,Y)}\Phi (T)-\bar{y}-\epsilon )\cap (C\setminus \{0\})=\emptyset$. Therefore, $\bar{y}$ is *ϵ*-efficient point of (VD).

**Theorem (*ϵ*-strong duality)**

Let $\epsilon \in C,\bar{x}\in S$, and $0\in G(\bar{x})$. Suppose that the following conditions hold:

- $\left(\bar{x},\bar{y}\right)$ is an  *ϵ*-Henig properly efficient element of (VP);
- $\bar{I}(x)$ is generalized *C* × *D*-subconvexlike on *A*, where $\bar{I}(x)=(F(x)-\bar{y}+\epsilon )\times G(x)$;
- *vcl*(*cone*(*G*(*A*) + *D*)) = *Z*.

Then,$\;\bar{y}\;$is  *ϵ*-efficient point of (VD).

**Proof**

According to Lemma 15, there exists $\bar{T}\in L^{+}(Z,Y)$ such that $\left(\bar{x},\bar{y}\right)$ is an  *ϵ*-Henig properly efficient element of ${\left(\text{UVP}\right)}_{\bar{T}}$. Since $0\in G(\bar{x})$, we have

(36) $$\begin{array}{c} \bar{y}\in \epsilon -H_{\min}\left(\underset{x\in A}{⋃}L\left(x,\bar{T}\right),B\right)=\Phi \left(\bar{T}\right)\subseteq \underset{T\in L^{+}\left(Z,Y\right)}{⋃}\Phi \left(T\right). \end{array}$$

Since$\;\bar{y}\in F(\bar{x})$, it follows from Theorem 19 that $\bar{y}$ is *ϵ*-efficient point of (VD).

## 5. Conclusions

Based on [7], we introduce the concept of *ϵ*-Henig saddle point of the set-valued map in linear spaces. The relationships between the *ϵ*-Henig saddle point of the set-valued map and the *ϵ*-Henig properly efficient element of the set-valued optimization problem are established. Some duality theorems are obtained in the sense of *ϵ*-Henig proper efficiency. When *ϵ*-Henig proper efficiency is replaced by *ϵ*-super efficiency in linear spaces, whether the conclusions of this paper hold is an interesting topic.
